# Targeting PRKDC activates the efficacy of antitumor immunity while sensitizing to chemotherapy and targeted therapy in liver hepatocellular carcinoma

**DOI:** 10.18632/aging.205855

**Published:** 2024-05-22

**Authors:** Yitong Pan, Qiyao Zhu, Ting Hong, Jun Cheng, Xinhui Tang

**Affiliations:** 1 The Affiliated Cancer Hospital of Xiangya School of Medicine, Central South University/Hunan Cancer Hospital, Changsha 410013, Hunan, China; 2 CAS Key Laboratory of Genomic and Precision Medicine, Beijing Institute of Genomics, Chinese Academy of Sciences, Beijing 100101, China; 3 Department of Spine Surgery, The Third Xiangya Hospital, Central South University, Changsha 410013, Hunan, China

**Keywords:** programmed cell death, liver hepatocellular carcinoma, tumor microenvironment, immunotherapy, PRKDC

## Abstract

Background: Liver hepatocellular carcinoma (LIHC) ranks among the malignancies with the highest mortality rates, primarily due to chemoresistance culminating in treatment failure. Despite its impact, predictive models addressing disease progression, tumor microenvironment, and drug sensitivity remain elusive for LIHC patients. Recognizing the significant influence of various programmed cell death (PCD) modes on tumor evolution, this study investigates PCD genes to elucidate their implications on the prognosis and immune landscape of LIHC.

Methods: To develop the classification and model, we employed a total of 17 genes associated with PCD patterns. To collect data, we acquired gene expression profiles, somatic mutation information, copy number variation data, and corresponding clinical data from the TCGA database, specifically from LIHC patients. Moreover, we obtained spatial transcriptome data and additional bulk datasets from the Gene Expression Omnibus (GEO) database to conduct further analysis. Various experiments were conducted to validate the role of the PCD gene PRKDC in proliferation, migration, invasion, EMT, cell cycle, therapeutic sensitivity, and antitumor immunity.

Results: A novel LIHC classification based on these genes divided patients into two clusters, C1 and C2. The C2 cluster exhibited characteristics indicative of poor prognosis and an immune-activated microenvironment. This group showed greater response potential to immune checkpoint inhibitors, displaying higher levels of certain immune signatures and receptors. A programmed cell death index (PCDI) was constructed using 17 selected PCD genes. This index could effectively predict patient prognosis, with higher PCDI indicating poorer survival rates and a more pro-tumor microenvironment. Immune landscapes revealed varying interactions with PCDI, suggesting therapeutic targets and insights into treatment resistance. Moreover, experiments results suggested that PRKDC can augment the invasive nature and growth of malignant cells and it can serve as a potential target for therapy, offering hope for ameliorating the prognosis of LIHC patients.

Conclusions: The study uncovers the insights of programmed cell death in the prognosis and potential therapeutic interventions. And we found that PRKDC can serve as a target for enhancing the efficacy of antitumor immunity while sensitizing chemotherapy and targeted therapy in liver hepatocellular carcinoma.

## INTRODUCTION

Liver hepatocellular carcinoma (LIHC), a third most common cause of cancer-related deaths worldwide, remains a significant global health concern [1]. With rising incidence rates, it is imperative to understand the molecular characteristics and genetic predispositions that govern its progression and influence patient prognosis. The challenge is further compounded by the myriad of cellular processes that contribute to its pathogenesis, rendering the identification of viable therapeutic targets and prognostic markers vital for improving patient outcomes.

One such crucial cellular process is programmed cell death (PCD). Unlike accidental cell death, which is uncontrolled, PCD is characterized by a complex array of regulated mechanisms [2]. Within the realm of PCD, several types have been identified, each with its distinct molecular signatures and functional implications. Apoptosis is an orderly cell death process where cells are removed without causing inflammation. Necroptosis, initially thought to be uncontrolled, is a form of programmed necrosis marked by the assembly of necrosomes. Pyroptosis is an inflammatory mode of cell death resulting in cell lysis and pro-inflammatory factor release. Ferroptosis involves iron-dependent lipid peroxidation leading to cell death, while the recently identified cuproptosis stems from copper-triggered cell demise. Entotic cell death, a non-apoptotic pathway, sees one cell actively engulfing another. Netotic cell death results from the release of neutrophil extracellular traps (NETs), often in response to stress including infection or injury. Parthanatos is triggered by overactivation of the PARP-1 nuclease, and lysosome-dependent cell death occurs when hydrolases permeate into the cytosol after membrane rupture. Autophagy-dependent cell death employs lysosomal degradation, aiding in adaptation of metabolism as well as nutrient cycling. Alkaliptosis is a newly characterized PCD form influenced by cellular alkalinization, and oxeiptosis integrates reactive oxygen sensing mechanisms, potentially co-regulating other cell death pathways [3].

The evolving understanding of PCD has not only provided insight into cellular dynamics but has also paved the way for therapeutic developments [2]. Drugs like BCL-2 inhibitors, approved for specific malignancies, manipulate the apoptosis pathway, while others target novel PCD forms, holding promise for future treatment regimens [4]. Additionally, those PCD also are implicated in the tumor microenvironment and immunotherapy [5].

In the landscape of LIHC, the role and impact of PCD remain under-explored. Given the pivotal role of PCD in cancer progression, tumor microenvironment and drug resistance, a thorough comprehension of its influence in LIHC is of paramount importance. Recognizing survival-associated genes through comprehensive genomic databases could offer valuable prognostic insights and guide tailored therapeutic strategies. Consequently, our study endeavors to establish a cell death index (CDI) for LIHC, aiming to predict therapeutic efficacy and prognosis. In essence, our investigation seeks to unravel the heterogeneity within LIHC, assess clinical outcomes, and potentially aid in optimizing therapeutic choices for patients.

## MATERIALS AND METHODS

### Aggregation of transcriptome and spatial transcriptomic datasets

The TCGA data portal (https://portal.gdc.cancer.gov/) provided access to transcriptome datasets and clinical data of liver cancer patients, including Stage T stage, overall survival (OS), disease-specific survival (DSS), and disease-free survival (DFS). (https://portal.gdc.cancer.gov/) [6]. GEO datasets (GSE14520, GSE76427, GSE116174, and GSE144269) were acquired from the Gene-Expression Omnibus (GEO) for the purpose of verification. These datasets were obtained to validate the findings and conclusions drawn from the study. Furthermore, spatial transcriptomic datasets from three different liver cancer patients were also obtained from the work conducted by Gu et al. These datasets are crucial for further analysis and comparison, enabling comprehensive research in the field of liver cancer [7]. To perform the bioinformatics analysis, we utilized R Bioconductor packages and R 4.1.1. The webpage https://gdc.cancer.gov/about-data/publications/panimmune provides information on the T cell receptor/B cell receptor (TCR/BCR) richness in liver cancer samples.

### Quantification of immune cell infiltration and evaluation of immunotherapy outcomes

Thorsson et al. published the findings on the Fraction of Leukocytes, Heterogeneity within the Tumor, Impaired Homologous Recombination, Shannon Diversity of BCR/TCR, and Richness of BCR/TCR in patients with TCGA LIHC [8]. To evaluate the distribution of immune cells within the tumor, we utilized the CIBERSORT algorithm [9], available for download from the Tumor Immune Estimation Resource (TIMER2.0) database [10]. Furthermore, the TIDE score and immunotherapy outcomes were computed using the TIDE website (http://tide.dfci.harvard.edu/) [11].

### Calculation of immune signatures

In order to investigate the immune profiles of individuals diagnosed with liver cancer, we collected an additional set of 17 genes associated with immune function. This gene set encompasses MHCI, MHCII, and pro-tumor cytokines, among other factors [12].

### Gene set variation analysis (GSVA) analysis

In order to examine the differences in immune associated signatures and HALLMARK among individuals with liver cancer, we conducted GSVA enrichment analysis using the ‘GSVA’ R package [13]. The gene set for HALLMARK was sourced from the MSigDB database (http://software.broadinstitute.org/gsea/msigdb/index.jsp). Pathway enrichment analysis was performed utilizing the fgsea package [14].

### Establishing classification and machine learning models utilizing programmed cell death genes

Zou et al. compiled a comprehensive list of 1078 genes that are involved in programmed cell death (PCD), including genes that regulate twelve different PCD patterns [3]. To identify differentially expressed genes (DEGs) related to PCD, we used the Limma package [15]. DEGs were identified using a criterion of adjusted p-value less than 0.05 and an absolute value of log2 fold change greater than 1. Additionally, we performed univariate Cox proportional hazard regression to identify PCD genes that may be associated with the prognosis of liver cancer. Based on their analysis, a total of 83 PCD genes were selected for further investigation. These genes showed promising associations with the progression and prognosis of liver cancer patients. This finding suggests that these PCD genes may play a crucial role in the development and clinical outcomes of liver cancer.

Additionally, using the R package ‘NMF’ [16], liver cancer patients were categorized into two cohorts depending on selected PCD genes. Then, by employing the machining learning algorithm and incorporating these PCD-related genes, a reliable model was constructed to forecast the prognosis of patients with liver cancer.

Further, we utilized the least absolute shrinkage and selection operator (LASSO) regression [17] analysis to reduce dimensionality and identify optimal variables. These variables, along with their regression coefficients, were used to calculate the PCDI. Patients were then divided into high- and low-PCDI scores groups based on the median value.

### Prediction of drug sensitivity among groups with varying PCDI levels

In the study reported by reference, we utilized the calcPhenotype function from the R package ‘oncoPredict’ to predict the half maximal inhibitory concentration (IC_50_) of drugs. To determine favorable drugs, a p-value threshold of 0.05 was employed. This selection criterion was based on gene expression profiles obtained from various cell lines [18].

### Cell culture and cell transfection

Huh-7 (LIHC), Hep3B (LIHC) and other cells were obtained from Xiangya Medical College Cell Bank (Changsha, China). Cell lines were grown in DMEM containing 10% fetal bovine serum. shRNAs were sourced from Genechem, Shanghai, China. Following the suggested guidelines, the cells underwent transfection and underwent selection with 2 ug/mL puromycin from Beyotime, Shanghai, China. The shRNA-PRKDC sequences were:

shPRKDC#1: Forward 5'-GCCTTACTAGAAGCTATATTG-3

Reverse 5’-CAATATAGCTTCTAGTAAGGC-3

shPRKDC#2: Forward 5’-CCTGAAGTCTTTACAACATAT-3

Reverse 5’-ATATGTTGTAAAGACTTCAGG-3

shCon: Forward 5’-AATACGGCGATGTGTCAGG-3

Reverse 5’-CCTGACACATCGCCGTATT-3

### Western blot analysis and quantitative real-time PCR

The experimental method has been elaborated upon in an earlier study [19, 20]. For Western blot, RIPA was used to lyse cells and BCA kit was utilized to detect the protein level, then proteins were isolated in SDS-PAGE and then transferred to PVDF membranes before incubating with primary antibodies and second antibodies, finally, the membranes were visualized using an ECL system after soaking in luminescent buffer. The antibodies used for Western blot analysis were: β-Actin (1:5000; Cell Signaling Technologies, USA), PRKDC (1:2000; Proteintech, USA), Cleaved Caspase-3 (1:1000; Cell Signaling Technologies), Bax (1:1000; Cell Signaling Technologies), Bcl-2 (1:1000; Cell Signaling Technologies), E-Cadherin (1:1000; Cell Signaling Technologies), N-Cadherin (1:1000; Cell Signaling Technologies), and Vimentin (1:1000; Cell Signaling Technologies).

For quantitative real-time PCR, the RNA was extracted from cell with TRIzol, and the PrimeScript RT reagent kit was used to reverse transcribe. qRT-PCR was done using SYBR Premix ExTaq on an Applied Biosystems 7300 System. The mRNA primers for quantitative real-time PCR included:

PRKDC: Forward 5’-CATGGAAGAAGATCCCCAGA-3

Reverse 5’-TGGGCACACCACTTTAACAA-3

GAPDH: Forward 5’-GGAGCGAGATCCCTCCAAAAT-3

Reverse 5’-GGCTGTTGTCATACTTCTCATGG-3

CCL2: Forward 5’-TCGCGAGCTATAGAAGAATCA-3

Reverse 5’-TGTTCAAGTCTTCGGAGTTTG-3

CCL4: Forward 5’-CCAAACCAAAAGAAGCAAGC-3

Reverse 5’-ACAGTGGACCATCCCCATAG-3

CCL5: Forward 5’-GAGTATTTCTACACCAGTGGCAAG-3

Reverse 5’-TCCCGAACCCATTTCTTCTCT-3

CXCL1: Forward 5’-ACCCAAACCGAAGTCATAGCC-3

Reverse 5’-TTGTCAGAAGCCAGCGTTCA-3

CXCL8: Forward 5’-TTTTGCCAAGGAGTGCTAAAGA-3

Reverse 5’-AACCCTCTGCACCCAGTTTTC-3

CXCL10: Forward 5’TGGCATTCAAGGAGTACCTCTC-3

Reverse 5’-GGACAAAATTGGCTTGCAGGA-3

### Cell viability, clonogenic assay, wound healing assay, and transwell assay

For cell viability assay, 5000 cells were cultured in each well of 96-well plate overnight, then treated them using different reagents for a proper time, and detected the absorbance value at 460 nm after cultured with 10% CCK-8 reagent for 2 hours. For clonogenic assay, 500 cells were cultured in each well of 6-well plate overnight, and treated them using CD8+ T cells, after 2 weeks, the plates were scanned and clone number were counted. For wound healing assay, cells were seeded in 6-well plates, then wounded with a 200 μl tip when they reached 90% saturation, and photographed at 0 and 48 hours. For transwell assay, 4×104 cells in 2000 μl DMEM were seeded in the upper chambers, and 600 μl DMEM with 15% FBS were added to the lower chambers. 24 hours later, the cells were fixed and stained using crystal violet, and photographed using a microscope.

### Cell death analysis

Cells were plated at a suitable density in 12-well plates. Following the appropriate incubation time, they were gathered (inclusive of floating dead cells) and were stained with trypan blue. The proportion of dead cells was then measured using a microscope.

### Cell cycle analysis

Cell Cycle Analysis Kit from BD Biosciences, Shanghai, China was employed and the protocol provided was adhered to. Briefly, 2×106 transfected cells were fixed using 70% ethanol for 24 hours at 4° C, rinsed with PBS, and then stained with propidium iodide in darkness for 30 minutes. Subsequently, flow cytometric detection was conducted. The results were interpreted using ModFit LT 5.0.

### ELISA

Supernatants from cell cultures in 24-well plates were harvested, and the chemokines including CCL2, CCL4, CCL5, CXCL1, CXCL8, and CXCL10 were identified with ELISA kit (Proteintech, USA). Measurements were taken with a Biotech microplate reader (Thermo Fisher Scientific, USA) as per the given instructions. Chemokine concentrations were determined based on OD readings at 450 nm.

### CD8+ T cell migration assay

CD8+ T cells were extracted from human peripheral blood utilizing the Human CD8 T Cells Kit (#11348D, Thermo Fisher Scientific, USA) following the provided guidelines. The migration assay for these T cells was then conducted using a 24-well transwell system featuring a 6.5 mm diameter and 8 μm pore size polycarbonate membrane (Corning, USA). Supernatants, amounting to 600 μL, either from the PRKDC knockdown group or the control group of HCC cells, were added to the bottom chamber. Concurrently, 1 × 105 CD8+ T cells in 100 μl media were placed in the top chamber. Post a 6-hour incubation, the migrated T cells in the bottom chamber were gathered and quantified via a cell counting plate.

### T cell-mediated tumor cell-killing assay

HCC cells were plated in a 12-well plate and left overnight before a 48-hour co-culturing with T cells. Post-culturing, the cells were rinsed with PBS twice, and residual cells were stained with crystal violet. Images of these plates were captured, and subsequent OD readings were acquired at 570 nm using a microplate reader.

### Statistical analysis

The ‘maftools’ R package [21] was utilized to investigate masked somatic mutation data among LIHC patients. The Wilcoxon test was employed to compare multiple features among different groups. Spearman's correlation coefficient was used to calculate the correlation between the score of PCD and immune features combined with drug IC_50_. The ‘survminer’ R package [22] was used to apply Kaplan-Meier survival analysis to investigate the relationship between liver cancer patient groups and survival. Univariate Cox proportional hazard regression was utilized to examine the relevance between PCD index and overall survival, while multivariate Cox regression was employed to evaluate the independent prognostic significance of PCD index compared to other clinical parameters. A two-way ANOVA test was used to determine the effect of PRKDC and T cell on cell survival. *P*-value below 0.05 was considered statistically significant.

### Availability of data and materials

We utilized the liver cancer (LIHC) dataset from the TCGA repository (https://portal.gdc.cancer.gov/projects/TCGA). We also acquired four validation LIHC datasets, namely GSE14520, GSE76427, GSE116174, and GSE144269 from the publicly available GEO database (http://www.ncbi.nlm.nih.gov/geo). In addition, we retrieved the raw data of spatial transcriptomic dataset from three liver cancer patients from Gu et al. Both datasets can be accessed freely for research use.

## RESULTS

### Genomics and functional landscapes of prognosis associated programmed-cell-death-genes in LIHC patients

In our study, we exploited the TCGA-LIHC cohort and applied the Limma algorithm to identify 815 differentially expressed PCD genes from 17 PCD patterns. The selection criteria included an adjusted p-value less than 0.05 (Supplementary Figure 1A). We then used the unique Cox method to analyze the identified genes and found that 100 of them were significantly correlated with the prognosis of LIHC patients. By intersecting the results, we identified a total of 83 PCD genes that were relevant to OS or tumor progression (Supplementary Figure 1B). To further confirm the universal expression of the PCD genes, we included four GEO datasets related to liver cancer (GSE14520, GSE76427, GSE116174, and GSE144269) in our investigation. After conducting a comprehensive intersection analysis, we found that 45 PCD genes showed consistent expression patterns in multiple datasets. Therefore, we considered these genes appropriate for further analysis (Supplementary Figure 1C). The Gene Ontology (GO) enrichment analysis indicated that the differentially expressed genes (DEGs) are linked to PCD and apoptotic-related pathways, including extrinsic apoptotic signaling pathway, intrinsic apoptotic signaling pathway, regulation of autophagy, and regulation of apoptotic signaling pathway, demonstrating that the DEGs plays a pivotal role in regulating the process of cellular demise. Extrinsic and intrinsic apoptotic signaling pathways directly participate in the programmed process of cell death, responding to death signals originating from extracellular and intracellular sources, respectively. Additionally, the regulation of autophagy modulation and apoptotic signaling pathways further signifies the ability of cells to finely regulate autophagy and apoptosis mechanisms to maintain cellular homeostasis or promote orderly cell death in response to adverse conditions (Supplementary Figure 1D). Additionally, Kyoto Encyclopedia of Genes and Genomes (KEGG) analysis highlighted the DEGs’ role in crucial biological pathways such as mTOR, JAK-STAT, and HIF-1 signaling (Supplementary Figure 1E). The study also evaluated the variation in PCD-related genes and found that approximately 54.08% (199/368) of LIHC patients had mutation sites, with TP53 having the highest mutation frequency of 29% (Supplementary Figure 1F, 1G). Additionally, the CNV status of these 45 PCD genes was displayed in Supplementary Figure 1H.

### Establishment of a novel classification of LIHC based on cell death-related genes

**Figure 1:**
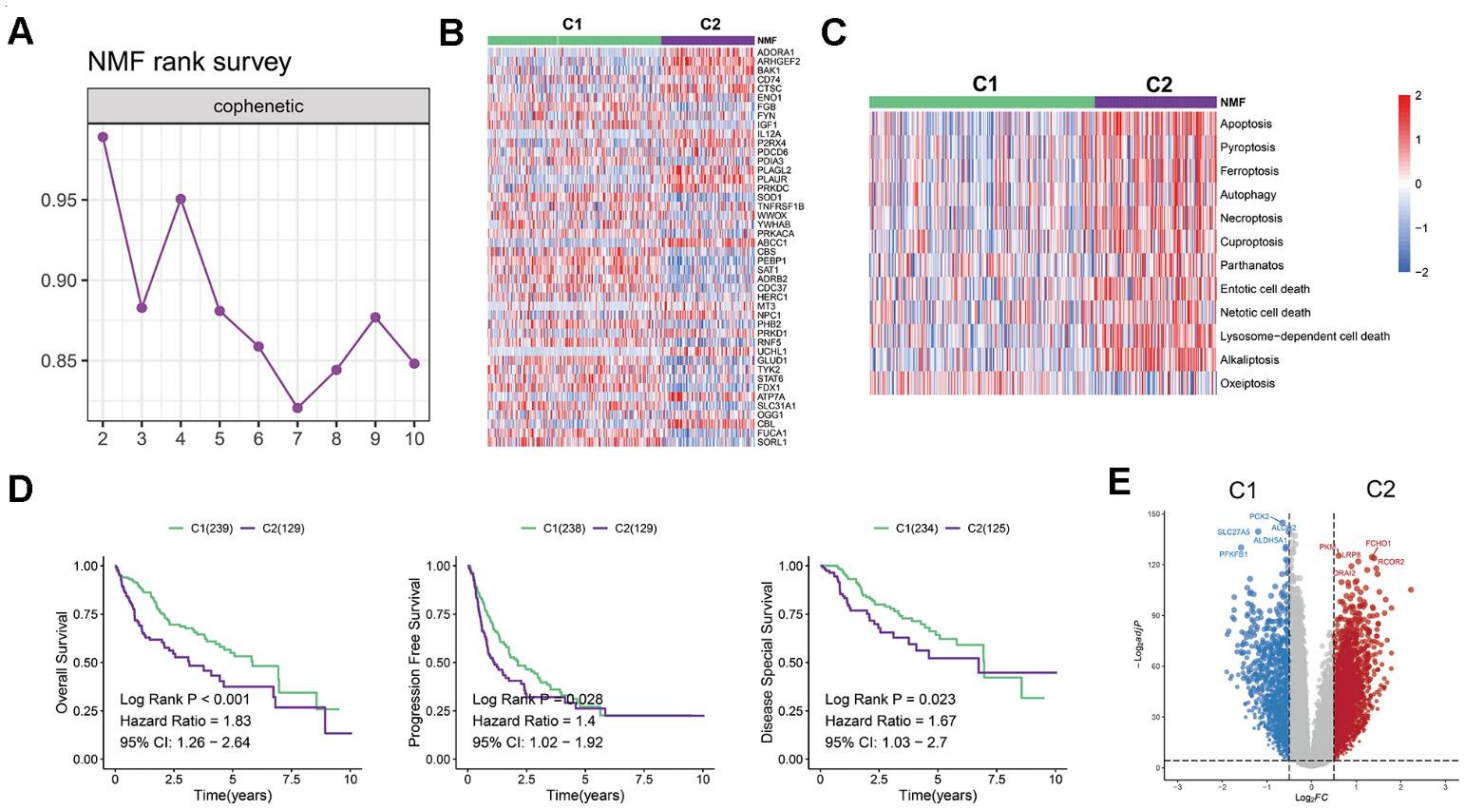
**Establishment of a novel LIHC classification based on cell death-related genes.** (**A**) LIHC patients were classified into two molecular groups using NMF algorithm. (**B**) Heatmap displays the expression of 45 selected PCD genes in the two groups. (**C**) Heatmap shows the enrichment scores of cell death patterns between the clusters. (**D**) Kaplan-Meier analysis reveals the overall survival, progression free survival rates, and disease-spatial survival for cluster 1 and 2 groups, demonstrating superior prognoses for patients in cluster 1 compared to those in cluster 2. (**E**) Volcano plot illustrates differentially expressed genes (DEGs) in the two clusters.

To uncover potential subtypes of liver hepatocellular carcinoma (LIHC) patients, we conducted a non-negative matrix factorization (NMF) analysis using 45 prognosis-related PCD genes. Our findings suggest that LIHC patients can be classified into two distinct clusters, namely C1 and C2, when n=2. This was demonstrated by the pronounced distinctions between the subgroups (Figures 1A). The expression of the 45-prognosis related PCD genes in the two clusters was displayed in Figure 1B. We also investigated the enrichment scores of cell death patterns between these, observing that the scores of C2 patients were more pronounced than those of cluster 1 (Figure 1C). Additionally, distinct differences in overall survival (OS), progression-free interval (PFS), and disease-spatial survival (DSS) were observed between the two clusters (P < 0.05, Figure 1D). While cluster 1 showed a favorable prognosis, cluster 2 was indicative of a poorer outcome. To delve into possible mechanisms, we contrasted mRNA expression between the C1 and C2 groups (Figure 1E). Employing the clusterProfiler R package, it was discerned that cluster 2 was enriched in pathways tied to tumor metastasis and progression, such as the Hippo, PI3K-Akt, TGF-beta, and NF-kappa B signaling pathways (Supplementary Figure 1I, 1J).

In contrast, the cluster 1 showed an up-regulation of metabolic pathways (e.g., glutathione metabolism, tryptophan metabolism, or fatty acid metabolism) as shown in Figures 1F. The results of hallmark pathway enrichment also confirmed that C2 patients enriched in pathways linked to tumor cell proliferation, including MITOTIC_SPINDLE, MYOGENESIS, E2F_TARGETS, and G2M_CHECKPOINT (Supplementary Figure 1K).

### Immunotherapy showed greater efficacy in LIHC patients categorized in C2 group

Considering the pivotal role of immunotherapy as a therapeutic strategy for cancer, coupled with the substantial impact of cell death in triggering antitumor immune responses, we examined the tumor microenvironment (TME) in both clusters.

**Figure 2:**
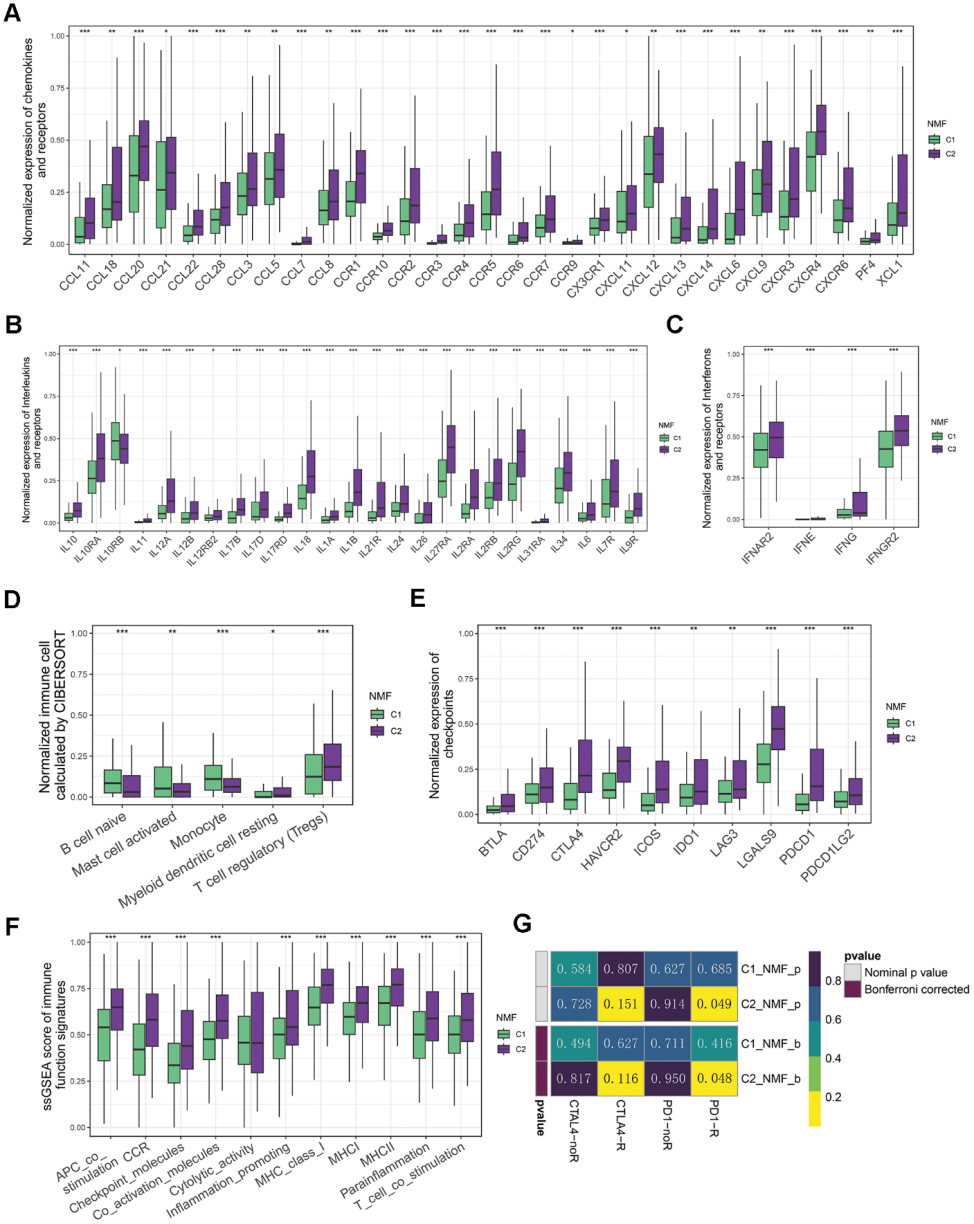
**Immune landscapes of two groups.** (**A**) Comparative box plots illustrating normalized expression levels of chemokines and receptors in two groups. (**B**) Comparative box plots showcasing normalized expression levels of interleukins and their receptors in two groups. (**C**) Comparative box plots demonstrating normalized expression levels of interferons and their receptors in two groups. (**D**) Comparative box plots revealing normalized fraction levels of infiltrated immune cells calculated by CIBERSORT in the two clusters. (**E**) Comparative box plots displaying normalized expression levels of immune checkpoints in two groups. (**F**) Comparative box plots presenting ssGSEA scores for immune function signatures between two groups. (**G**) Submap result for predicting the immunotherapy of anti-CTLA4 and anti-PD1 in C1 and C2 groups (*P < 0.05, **P < 0.01, and ***P < 0.001 determined by Wilcoxon test).

As Tertiary lymphoid structures (TLS) act as germinal centers for immune cells in the TME, we assessed the expression of chemokines that contribute to TLS formation. Interestingly, we observed a high expression of most chemokines in C2. More specifically, C2 exhibited elevated levels of CCL11, CXCL13, CXCL9, CXCL11, CCL7, CCL20, CCR4, CCL18, CXCR4, CXCR3, CCR3, CCR7, CCR5, CCR2, CCL5, CCR6, CCR1, CCL3, CCL22, CCL8, XCL1, CXCR6, CCR9, PF4, CXCL6, CCR10, CX3CR1, CXCL14, CXCL12, CCL21, and CCL28 (Figure 2A). Furthermore, we observed that numerous interferons and their related receptors (e.g., IFNG, IFNAR2, IFNGR2), along with many interleukins and their receptors, were linked to immune response activation (Figure 2B, 2C). Consistently, we also found that C2 had a higher proportion of immune-stimulating cells, namely B cell naive, Mast cell activated, and Monocyte. Conversely, C1 had a larger proportion of immunosuppressive cells, including myeloid dendritic cell resting and regulatory T cells (Tregs) (Figure 2D).

**Figure 3:**
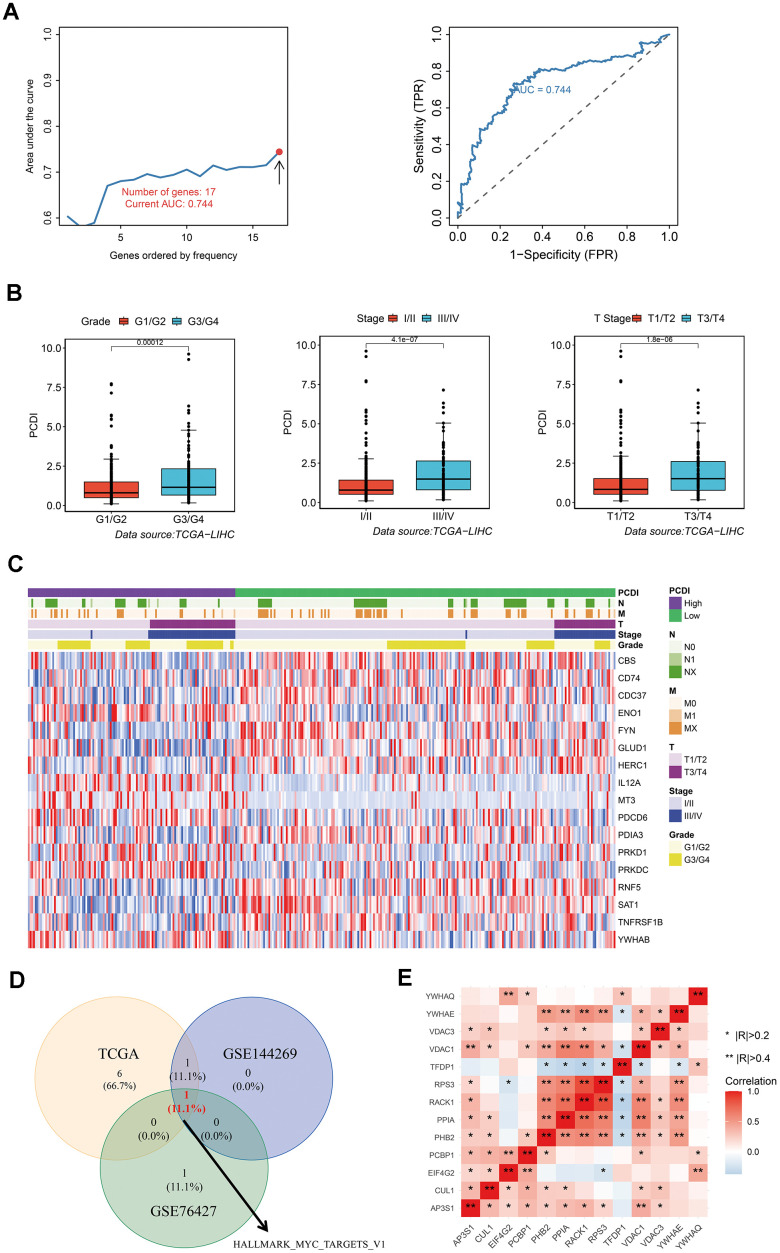
**Development of a reliable programmed cell death index (PCDI).** (**A**) A comparison of the AUC values for various models and the AUC of the final PCDI is presented. (**B**) Box plots demonstrate the correlation between PCDI and clinical features such as Grade, Stage, and T. (**C**) Heatmap displays the expression of PCD genes in the model. (**D**) Venn diagram illustrates the overlap of enriched hallmark pathways in three datasets (TCAG, GSE144269, and GSE76427). (**E**) Correlation analysis of genes within the HALLMARK_MYC_TARGETS_V1 pathway.

Considering the significance of the presence of immune checkpoints as a fundamental factor in immune checkpoint inhibitors (ICIs) treatment, we conducted a further analysis on the expression of immune checkpoints within 2 distinct clusters. Of note, the levels of numerous checkpoints (e.g., CD274/PD-L1, CTLA4, PDCD1/PD1, IDO1/2, LAG3, TIGIT, HAVCR2/TIM-3, PDCD1LG2/PD-L2) in C2 were higher than those in C1 (Figure 2E). In line with this, we found that C2 group also exhibited higher scores of immune function signatures, including MHCI, MHCII, and inflammation promoting etc. (Figure 3G). This disparity suggests that patients with LIHC in C2 exhibited a greater sensitivity to immunotherapy. Due to the unavailability of publicly accessible LIHC immunotherapy-related datasets, we employed the submap algorithm to predict the sensitivity of groups C1 and C2 to immunotherapy. Consistent with previous findings, the submap results demonstrated that C2 exhibited greater sensitivity to anti-PD1 immunotherapy, which was further confirmed by high score of TIDE in C2 (Figure 2G and Supplementary Figure 2A). Moreover, the results indicate that patients with the C2 group features exhibit higher levels of somatic diversification in their immunoglobulins or immune receptors, such as Leukocyte Fraction, Intratumor Heterogeneity, and Homologous Recombination Defects (Supplementary Figure 2B–2D). Additionally, the C2 group also shows an increase in TCR/BCR Shannon and Diversity (Supplementary Figure 2E, 2F).

Overall, the TME in C2 patients showed enriched TCR/BCR diversity as well as up-regulated expression of immune checkpoints, suggesting that these patients may benefit more from ICIs.

### Construction of a robust programmed cell death index for predicting the prognosis of LIHC patients

To establish a reliable PCD index for predicting the prognosis of LIHC patients, we utilized LASSO and multiple Cox regression analysis. We conducted 500 iterations mitigate the random errors associated with LASSO regression and ultimately selected 17 PCD genes. These genes include CBS, CD74, CDC37, ENO1, FYN, GLUD1, HERC1, IL12A, MT3, PDCD6, PDIA3, PRKD1, PRKDC, RNF5, SAT1, TNFRSF1B, and YWHAB. We then used these genes to create a model and retained the model with the highest five-year AUC (Figure 3A). We observed that high PCDI patients were significantly associated with advanced stages such as Grade and Stage (Figure 3B). We generated a heatmap to display variances in gene expression profiles between TCGA LIHC patients with low and high PCDI, including their related clinical features (Figure 3C).

To discern variations in biological processes between the PCDI subgroups, we employed Gene set variation analysis (GSVA). Results from the three datasets (TCGA, GSE144269 and GSE76427) revealed that the HALLMARK MYC TARGETS V1 pathway was the most identified biological process (Figure 3D). Furthermore, the correlation between these genes in HALLMARK MYC TARGETS V1 pathway was displayed in Figure 3E.

### Exogenous verification of the precision and independence of PCDI

**Figure 4:**
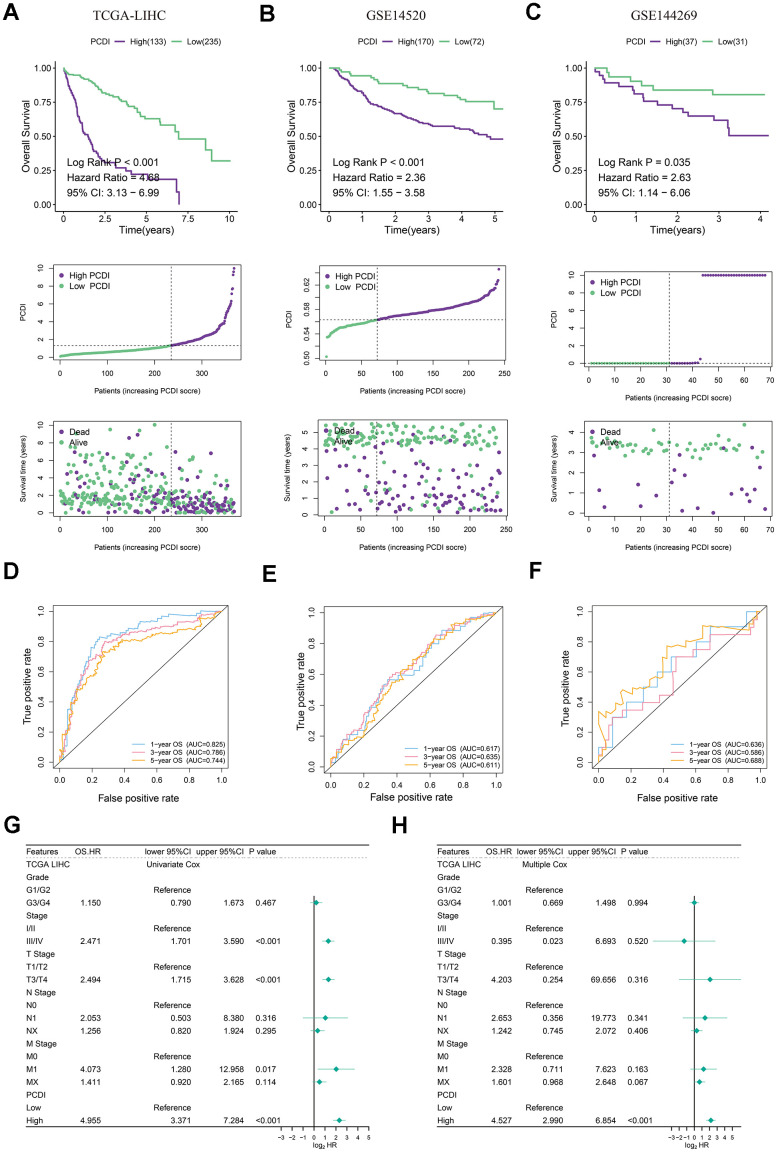
**Evaluation of exogenous datasets to assess the accuracy and independence of PCDI.** (**A**–**C**) Kaplan-Meier analysis of overall survival rates in high- and low-PCDI groups, indicating superior prognoses for patients with low PCDI scores compared to those with high PCDI scores. Additionally, the distribution of PCDI based on survival status and time is demonstrated in the three datasets (TCGA, GSE14520, and GSE144269). (**D**–**F**) Time-dependent ROC curve analysis of TCGA, GSE14520, and GSE144269 datasets. (**G**) Univariate Cox regression analysis revealing a significant correlation between PCDI and prognosis. (**H**) Multivariate Cox regression analysis confirming that PCDI can serve as an independent prognostic factor.

After establishing the PCDI, LIHC patients were categorized into low- and high-PCDI groups based on the specified PCD index cut-off calculated by survminer R package. Kaplan-Meier analysis indicated that those in the low PCDI category exhibited superior overall survival rates in both the TCGA and GEO datasets (P<0.05). Moreover, a heightened PCDI corresponded with shorted survival duration and diminished survival rates (Figure 4A–4C). We crafted time-dependent ROC curves in R to gauge the prognostic model’s predictive accuracy for both cohorts, evaluating the AUC at multiple intervals. The PCDI displayed robust reliability and prognostic potential, as the ROC curve demonstrated. In the training set, the 1-year AUC registered at 0.825, 3-year at 0.786, and 5-year at 0.744 (Figure 4D). For the validating GEO datasets, the 1-year AUC surpassed 0.6, 3-year exceeded 0.58, and the 5-year went beyond 0.6 (Figure 4E, 4F). Univariate Cox assessment highlighted an association between reduced survival and factors like pathological stage, pathological T, M stage, and elevated PCDI (Figure 4G). Conversely, the multivariate Cox assessment determined that solely the PCDI stood out as an independent prognostic determinant with a p-value below 0.001 (Figure 4H).

### PCD associated immune landscapes at bulk and two-dimensional spatial levels

**Figure 5:**
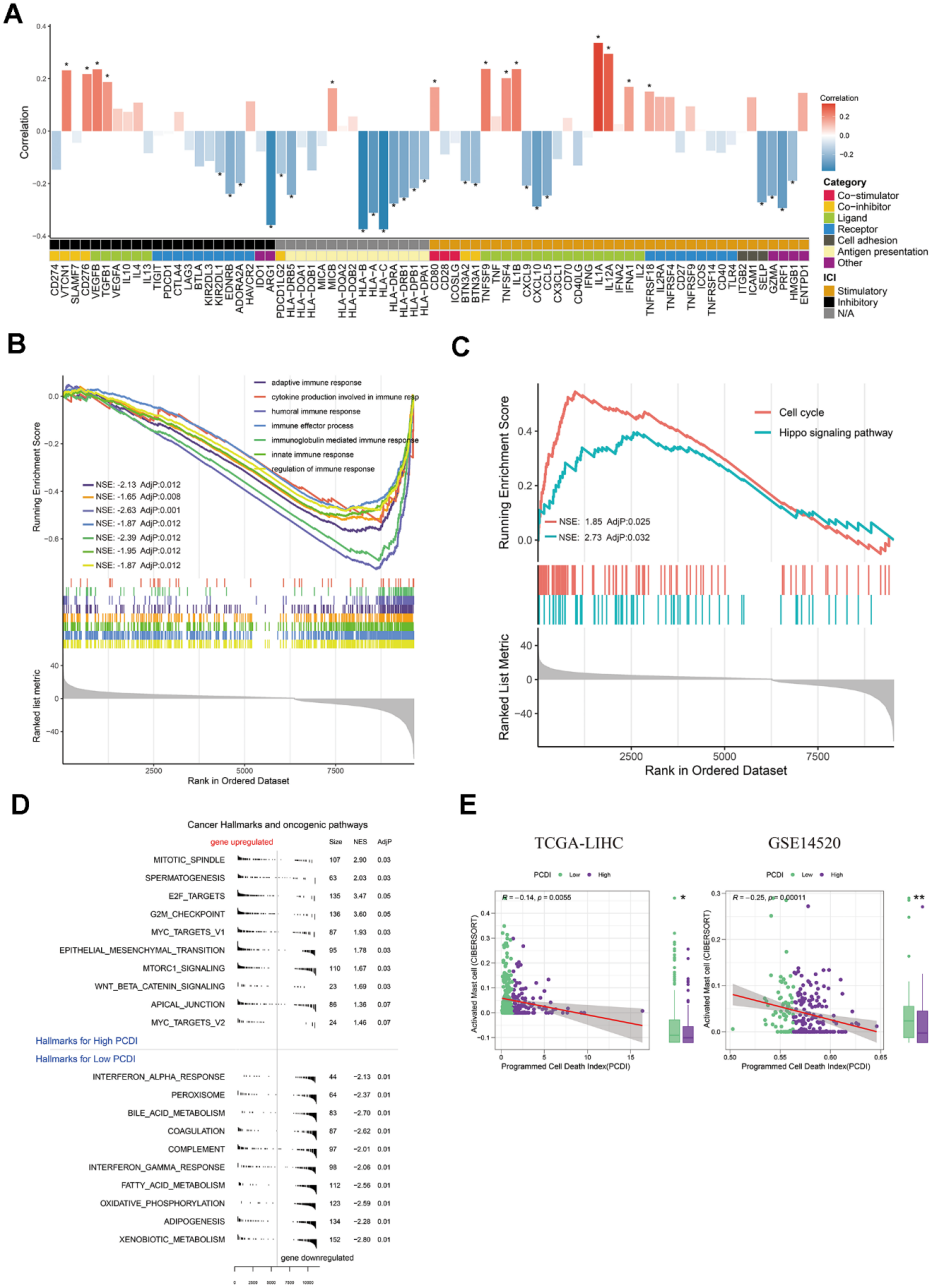
**Exploration of the potential mechanism of PCDI.** (**A**) Bar plot illustrating the correlation between immunomodulators and PCDI in the TCGA dataset. (**B**) GO enrichment analysis of immune response-related pathways in the two PCDI groups. (**C**) KEGG enrichment analysis of immune response-related pathways in the two PCDI groups. (**D**) Gene set enrichment analysis (GSEA) highlighting hallmark pathways in the two PCDI groups. (**E**) Correlation analysis between the fraction of activated Mast cells calculated by CIBERSORT and PCDI in the TCGA and GSE14520 datasets. (*P < 0.05, **P < 0.01, and ***P < 0.001 determined by Wilcoxon test).

Checkpoints and relevant modulators are considered as regulators in activation of immune cells in TME, thus we investigated the expression of immunomodulators engaged in infiltration of immune cells. We initially analyzed the correlation between immunomodulators and PCD index. Surprisingly, we discovered that low PCDI is positively associated with antigen presentation and receptors, whereas high PCDI is positively associated with co-inhibitors (CD276 and VTCN1) and ligand (TGFB1 and VEGFA) (Figure 5A). This indicates that a high PCDI might correlate with a pro-tumor microenvironment. Our hypothesis was confirmed by the GO and KEGG enrichment results. We observed that all signatures associated with immune response, including the humoral immune response, innate immune response, and adaptive immune response, were significantly downregulated in group with elevated PCDI. Conversely, aspects like the cell cycle and the Hippo signaling pathway saw increased activity in this group (Figure 5B, 5C). In line with this, Hallmark pathway enrichment highlighted the activation of pathways linked to therapy resistance and cancer cell invasion, such as MITOTIC SPINDLE, E2F TARGETS, G2M CHECKPOINT, MYC TARGETS V1, MTORC1 SIGNALING and EPITHELIAL MESENCHYMAL TRANSITION pathways were activated in high PCDI group (Figure 5D). Additionally, we observed a significantly higher PCDI score in the C2 group compared to the C1 group (Supplementary Figure 3A). Therefore, we hypothesize that the high PCDI group possesses immune characteristics consistent with those of the C2 group. To validate this hypothesis, we first computed the expression levels of interleukins and interferons in the high and low PCDI groups. Our findings revealed that the expression of interleukins, interferons, and their receptors was significantly higher in the high PCDI group compared to the low PCDI group (Supplementary Figure 3B, 3C). We also obtained fraction of immune cells calculated by CIBERSORT by using TIMER2.0 web server and we only found that the fraction of activated mast cell was negatively correlated with PCDI in multiple datasets (TCGA and GSE14520) (Figure 5E). To verify this result, we utilized the LIHC spatial transcriptome dataset to calculate the PCD index for each spot. Initially, PCDI was computed for each point using the PCD model. Simultaneously, to validate the spatial correlation between PCDI and Mast cells, we employed the GSVA algorithm to calculate the proportion of Mast cells at each location. Through Pearson correlation analysis, we found a negative correlation between PCDI and mast cell score in tumor regions (Supplementary Figure 4A–4C). This implies that mast cells may be inhibited in samples with high PCDI.

Taken together, these results provide a potential rationale for the decreased survival rate observed in high PCDI patients.

### Elucidating pharmacotherapeutic approaches for LIHC patients via PCD associated genes

**Figure 6:**
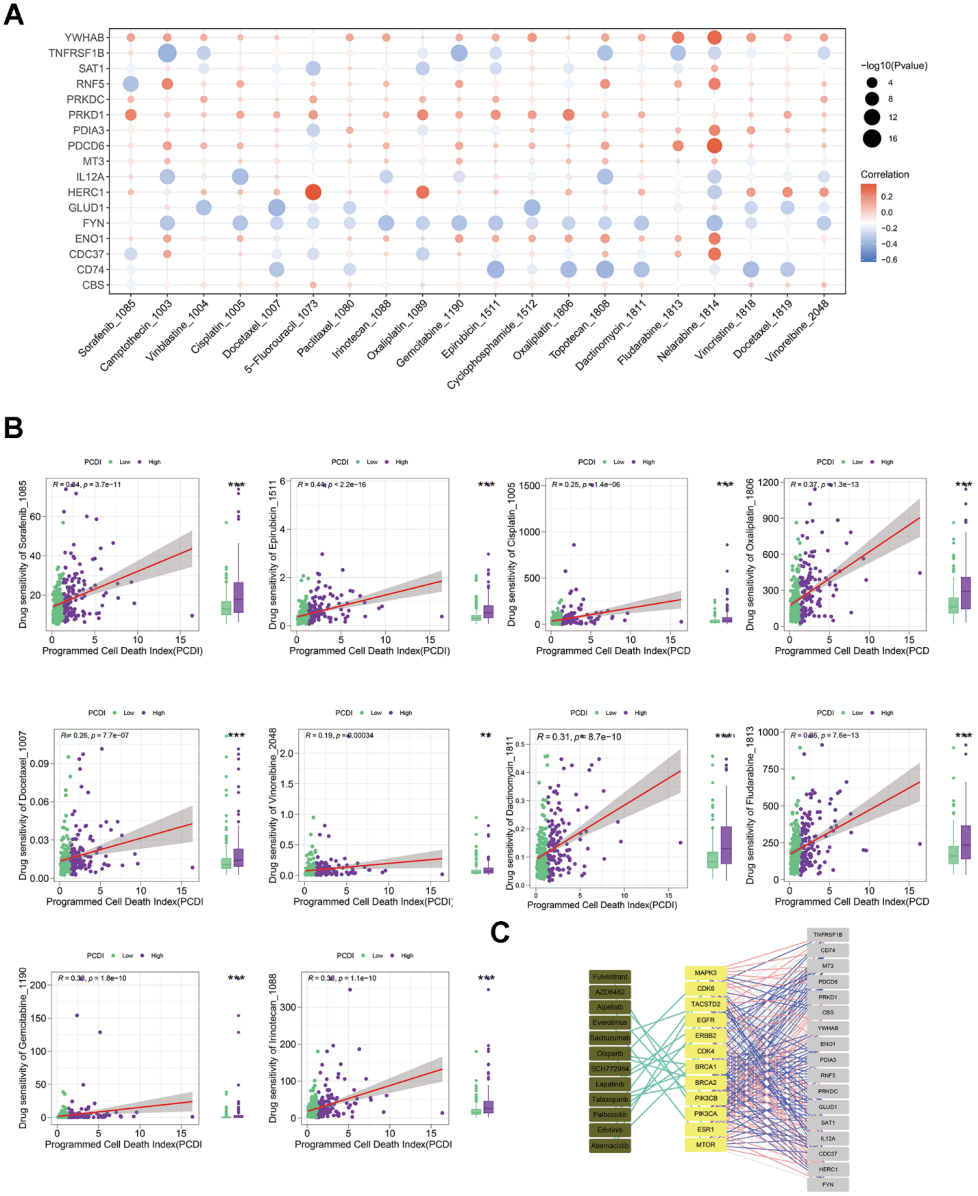
**Evaluating the predictive capability of the programmed cell death index for drug sensitivity.** (**A**) The correlation between the drug IC_50_ and PCD genes in model. (**B**) The correlation between the IC_50_ of drugs and PCDI values in patients with high- or low-PCDI, highlighting enhanced sensitivity to standard adjuvant chemotherapy in low PCDI LIHC patients. (**C**) The association between model genes and established targets for the treatment of LIHC.

To develop effective drug treatment strategies for LICH patients based on PCD features, we examined the IC_50_ values of drugs in LIHC samples to detect notable differences. Figure 6A depicts the relationship between drug sensitivities and genes in PCDI. We used the spearman correlation coefficient to select statistically significant drugs (P-value < 0.05 and R > 0.25). We found that LIHC patients in low-PCDI group exhibited greater vulnerability to chemical drugs (Dactinomycin, Fludarabine, and Gemcitabine), cell division inhibitors (Docetaxel and Vinorelbine), DNA damage drugs (Epirubicin and Irinotecan), and platinum drugs (Cisplatin and Oxaliplatin), as evidenced by the higher IC_50_ of tyrosine kinase inhibitor (Sorafenib) functional by inhibiting angiogenesis and interfering with tumor cell signaling (Figure 6B). In addition, those patients responded better to immunotherapy drugs, as evidenced by the elevated TIDE score in the high PCDI group (Supplementary Figure 2A). Figure 6C further illustrates the relationship between model genes and established therapeutic targets in LIHC.

Above all, these results indicate that liver cancer patients with low PCDI levels are potentially more responsive to immunotherapy combined with standard adjuvant chemotherapy treatments.

### PRKDC exhibits high expression in LIHC and enhances its proliferation, migration, and invasion

To further validate the efficacy of PCD model as a clinically applicable tool, we conducted further investigations on the genes associated with PCD in this model. Through univariate Cox regression analysis, we identified that among these genes, only 15 genes exhibited significant correlations with the prognosis of LIHC (Supplementary Figure 5A). Notably, Protein kinase, DNA-activated, catalytic subunit (PRKDC) was found to be specifically associated with the progression of tumor grade (Supplementary Figure 5B). The catalytic subunit of the DNA-dependent protein kinase (DNA-PK) is encoded by PRKDC. This kinase is a vital downstream effector in HKDC1-induced GC tumorigenesis, reliant on lipid metabolism [23]. While prior research indicated that PRKDC can enhance cell proliferation through the activation of MTORC [24], and it accelerates the proliferation and metastasis program in glioblastoma, colorectal cancer, gastric cancer, non-small cell lung carcinoma, nasopharyngeal carcinoma and osteosarcoma [25, 26]. However, its role in LIHC remains underexplored.

**Figure 7:**
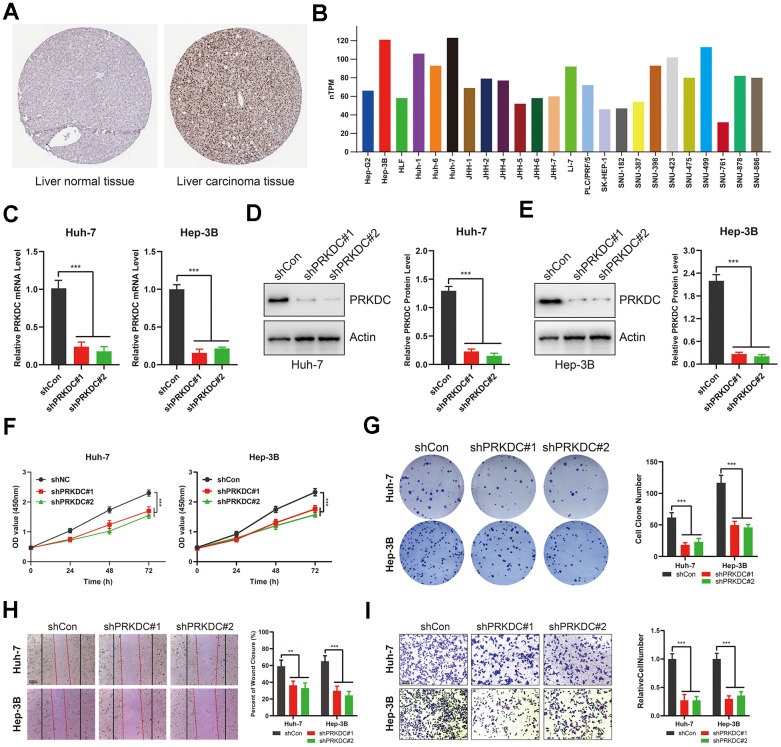
**PRKDC knockdown represses the invasiveness of LIHC cells.** (**A**) PRKDC expression in LIHC tissues from protein atlas. (**B**) PRKDC expression in LIHC cell lines from protein atlas. (**C**) Expression of PRKDC mRNA in Huh-7 or Hep-3B using qPCR. (**D**, **E**) Protein level of PRKDC in Huh-7 or Hep-3B using WB. (**F**) Cell viability in PRKDC knockdown and negative control groups. (**G**) Clone formation assays between PRKDC knockdown and negative control groups. (**H**) Would healing assays between PRKDC knockdown and negative control groups. (**I**) Relative migration of Huh-7 or Hep-3B cells between PRKDC knockdown and negative control groups.

Consistent with our bioinformatic findings, expression of PRKDC was markedly increased in LIHC tumor tissues relative to normal liver samples. (Figure 7A). This trend persisted at the cellular level: PRKDC expression was consistently high across LIHC cells, with either Huh-7 or Hep-3B cells showcasing the most pronounced expression (Figure 7B). Therefore, we generated PRKDC knockdown models in Huh-7 and Hep-3B cell lines using two distinct short hairpin (sh) RNAs. Their efficacy was confirmed through qPCR and Western blot analyses (Figure 7C–7E). Notably, PRKDC knockdown led to a marked inhibition of cell proliferation in both Huh-7 and Hep-3B cells (Figure 7F), suggesting a role for PRKDC in promoting LIHC cell proliferation. In line with this, the clonogenic capacity was diminished in PRKDC knockdown cells relative to their control counterparts (Figure 7G), hinting at PRKDC’s contribution to the unbounded growth potential of LIHC cells. Importantly, scratch and transwell migration assays revealed a decline in cell migration and invasion capabilities upon PRKDC depletion in both cell lines (Figure 7 H, 7I).

Collectively, these results underscore that PCD gene PRKDC fosters proliferation and invasion of LIHC, underlying the poor prognosis of LIHC patients with high-PCDI.

### PRKDC represses PCD, yet stimulates EMT and cell cycle arrest in LIHC

**Figure 8:**
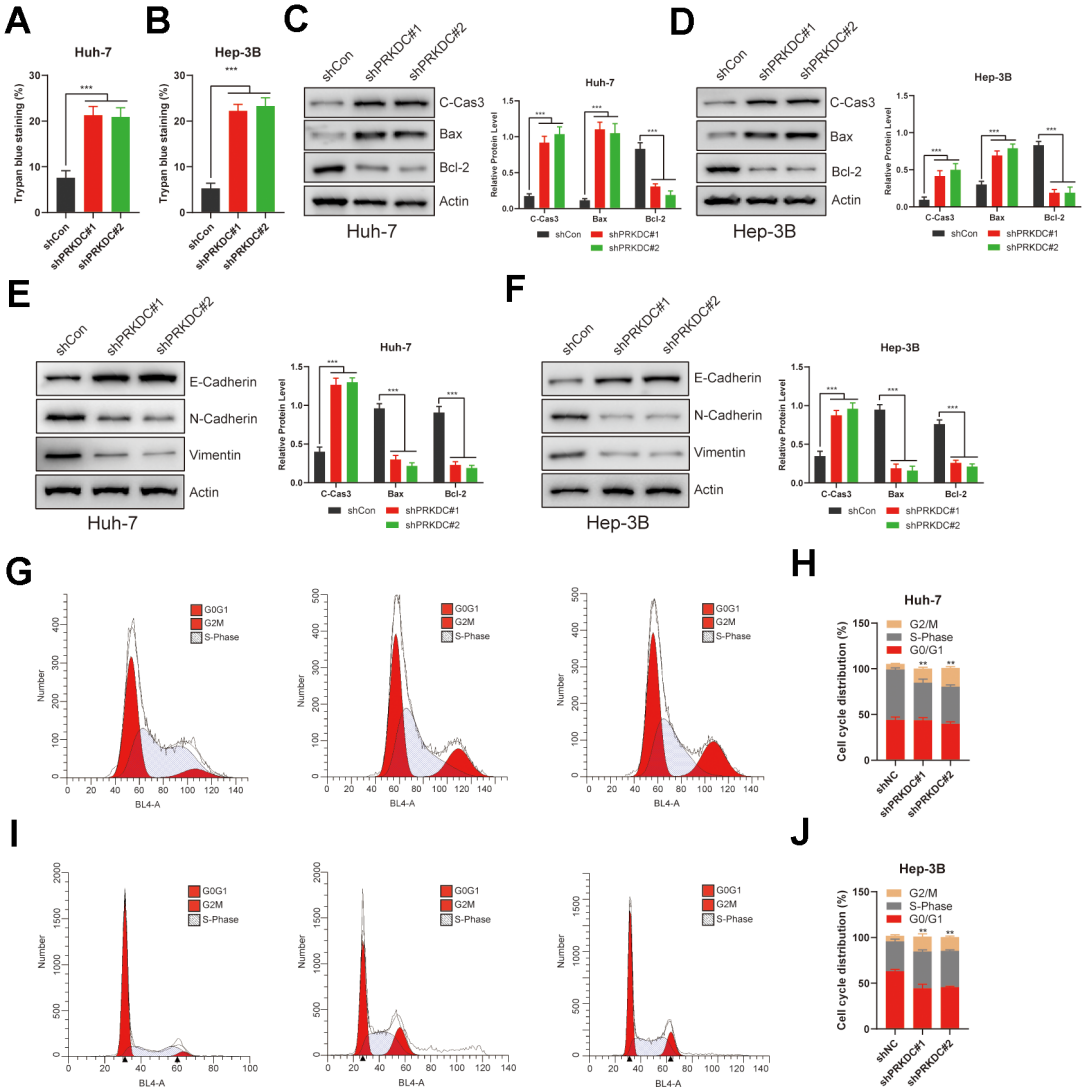
**PRKDC knockdown induced cell death, EMT, and halted the cell cycle in LIHC.** (**A**, **B**) Relative cell death percentages in Huh-7 or Hep-3B cells using trypan blue staining. (**C**, **D**) Protein level of apoptosis related markers in Huh-7 or Hep-3B cells. (**E**, **F**) Protein level of EMT related markers in Huh-7 or Hep-3B cells. (**G**–**J**) Cell cycle analysis between PRKDC knockdown and negative control groups in Huh-7 or Hep-3B cells using flow cytometry.

Given that PRKDC is a PCD gene, our initial experiments demonstrated that its knockdown promotes cell death, as evidenced by trypan blue staining (Figure 8A, 8B). Recognizing apoptosis as a prominent form of PCD, we evaluated apoptosis markers in Huh-7 and Hep-3B cells. Notably, pro-apoptosis markers such as cleaved caspase 3 and Bax were upregulated, while the anti-apoptosis marker Bcl-2 was downregulated in PRKDC knockdown cells. This pattern validates PRKDC’s role in suppressing PCD (Figure 8C, 8D). This inhibition of PCD partially elucidates the mechanism behind PRKDC-mediated augmentation of proliferation and invasion of LIHC cells.

The epithelial-mesenchymal transition (EMT) is a hallmark of enhanced invasiveness, migratory prowess, therapy resistance, and stem-like characteristics in cancer cells-all vital for cancer progression and metastasis. EMT also allows cancer cells to avoid apoptosis and stimulate tumor progression [27]. Consistent with this, PRKDC knockdown led to a rise in epithelial Cadherin expression but a decline in neural-type Cadherin and Vimentin in Huh-7 and Hep-3B cells (Figure 8E, 8F), suggesting that PRKDC promotes EMT process in LIHC. Furthermore, PRKDC knockdown induced G2/M phase arrest in the aforementioned cells, potentially underlying the observed growth restraint in these knockdown cells (Figure 8G–8J).

Overall, the combined effects of inhibiting PCD, augmenting EMT, and inducing cell cycle arrest underpin the enhanced proliferation and invasiveness seen in LIHC cells. This likely contributes to the poor prognosis observed in LIHC patients with high-PCDI.

### PRKDC inhibition potentiates antitumor immunity and sensitizes LIHC to chemotherapy and targeted therapy

**Figure 9:**
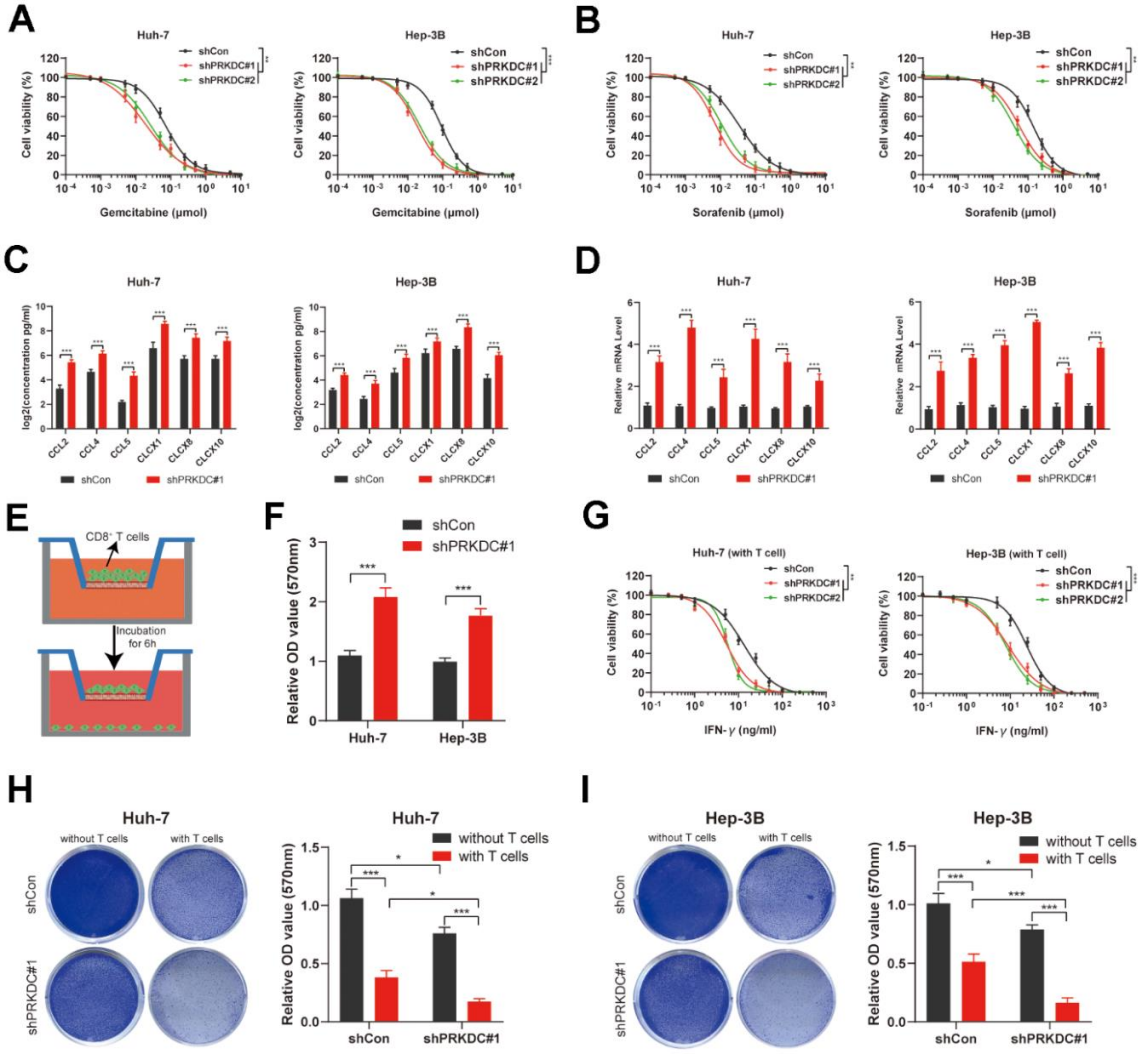
**PRKDC knockdown sensitizes Huh-7 and Hep-3B to chemotherapeutic and immunotherapeutic agents.** (**A**) Dose response curves of Gemcitabine. (**B**) Dose response curves of Sorafenib. (**C**, **D**) Chemokines including CCL2, CCL4, CCL5, CLCX1, CLCX8 and CLCX10 are detected using ELISA and qPCR. (**E**, **F**) Schematic representation of the CD8+ T cell migration experiment, along with the comparative migration of CD8+ T cells between the PRKDC knockdown and the control groups. (**G**) Dose response curves of IFN-γ in Huh-7 and Hep-3B co-cultured with CD8^+^ T cells. (**H**, **I**) Comparison of T cell-induced tumor cell death between PRKDC-silenced and control groups in Huh-7 or Hep-3B cells. (*P < 0.05, **P < 0.01, and ***P < 0.001 determined by two-way ANOVA test).

Our preliminary bioinformatic analyses suggest that patients exhibiting high PCDI show resistance to both chemotherapy and targeted therapy. Echoing this, knocking down PRKDC made Huh-7 and Hep-3B cells more responsive to Gemcitabine (a chemotherapeutic agent) and Sorafenib (a targeted therapeutic agent) (Figure 9A, 9B). This implies that PRKDC could be a potential sensitizing target for chemotherapy or targeted therapy in patients with high PCDI.

Importantly, our bioinformatic data implies a pro-tumoral microenvironment in patients with pronounced PCDI. Previous studies revealed that chemokines such as CCL2, CCL4, CCL5, CLCX1, CLCX8 and CLCX10 are important for global recruitment of immune cells [28]. In our study, ELISA and qPCR results further illuminated that the knockdown of PRKDC augmented the concentration and expression of cytokines, encompassing CCL2, CCL4, CCL5, CLCX1, CLCX8, and CLCX10, in the cell microenvironment (Figure 9C, 9D), lending weight to our bioinformatic findings. To define if PRKDC restrains infiltration of CD8^+^ T cells, we performed CD8+ T cell migration assay. The results indicated that PRKDC depletion potentiated CD8+ T permeation through the polycarbonate membrane (Figure 9E, 9F). Further, we co-cultured Huh-7 or Hep-3B cells with T cells to define PRKDC function in T cell-mediated antitumor immunity. Notably, the results showed that PRKDC knockdown heightened the sensitivity of Huh-7 and Hep-3B cells to CD8+ T cell-secreted IFN-γ (Figure 9G). Moreover, PRKDC knockdown not only decreased cell viability of Huh-7 or Hep-3B cells, but also amplified T cell-mediated cytotoxicity in both LIHC cell lines. These results suggest that PRKDC depletion increases the infiltration and antitumor effects of T cells (Figures 9H, 9I).

Together, PRKDC appears pivotal in LIHC cells’ resistance to chemotherapy and targeted treatments and fosters an immunosuppressive tumor microenvironment deterring antitumor immunity. As such, targeting PRKDC emerges as a potential avenue for optimizing chemotherapy, targeted therapy, and immunotherapy in LIHC patients characterized by high PCDI.

## DISCUSSION

The intricate and multifaceted relationship between programmed cell death (PCD) genes and liver hepatocellular carcinoma (LIHC) prognosis is an emerging research focus [29]. According to prior research, the role of cell death in antitumor immunity appears to be paradoxical [30]. Furthermore, the connection between cell death and TME, as well as its impact on TCA cycle, OXPHOS, and fatty acid metabolism, is intricately intertwined with antitumor immunity [31, 32]. In our study, a comprehensive approach was applied to discern this relationship, yielding findings that both underscore the pivotal role of PCD genes in LIHC prognosis and tumor microenvironment, and pave the way for potential immunotherapy, chemotherapy, and targeted therapy.

We initially discovered 815 differentially expressed PCD genes in the TCGA-LIHC dataset. Interestingly, a subset of 45 genes showcased consistent expression patterns across multiple liver cancer datasets. This strengthens the hypothesis that these genes play a non-negligible role in liver cancer progression and might be foundational for further investigation. The pathways they are involved in, like the mTOR and JAK-STAT signaling pathways, were implicated in various malignancies, attesting to their importance [33, 34]. One of the primary endeavors of our research was the development of a novel classification system for LIHC based on cell death-related genes. Our findings yielded two distinct clusters of patients. Strikingly, the two clusters had markedly different survival outcomes, with cluster 1 indicating a better prognosis compared to cluster 2. The underlying molecular mechanisms seem to revolve around differing pathways, while cluster 1 predominantly exhibited metabolic and complement pathways, cluster 2 was enriched in pathways linked to tumor progression. These molecular differences hint at potential therapeutic strategies tailored for each subgroup.

Hitherto, notable advancements have occurred in immuno-oncology, including the evolution of treatments like CPIs and CAR T cell therapy. These advancements hold great promise for overcoming tumors by activating the body’s own immune system [35]. However, there are still many patients who do not experience significant benefits from immunotherapy. Therefore, we investigated the response of these groups of patients to immunotherapy. The effectiveness of immunotherapy is well-established and involves multiple factors within a complex network. One crucial factor that influences immunotherapy efficacy is the TME, which is influenced by the presence and composition of tumor-infiltrating immune cells [36, 37]. These elements are pivotal in bolstering antitumor immunity. Notably, the C2 group, which was associated with a poorer prognosis, exhibited a more immune-activated microenvironment. One possible explanation is that the proportion of regulatory T (Treg) cells within group C2 is significantly higher than that within group C1, which indicates an abundance of immunosuppressive cells. As a distinct subset of T cells, Treg cells can dampen immune responses against foreign or self-antigens by suppressing effector T cells, mast cells, dendritic cells, and B cells, thereby maintaining immune tolerance within the body [38].

This observation is further corroborated by the cell proportion results calculated using CIBERSORT.

This notion is reinforced by the elevated expression of immune checkpoints in the C2 group, hinting at a more positive reaction to immune checkpoint inhibitors. Moreover, the C2 group exhibited a more pronounced presence of pro-tumor immune cells like resting myeloid dendritic cells and regulatory T cells (Tregs). In contrast, cluster 1 was characterized by a higher presence of anti-tumor immune cells such as naïve B cells, activated mast cells, and monocytes.

This was echoed in the increased levels of immune checkpoints in the C2 group, suggesting a more favorable reaction to ICIs. Additionally, the C2 group exhibited a more pronounced presence of pro-tumor immune cells like resting myeloid dendritic cells and Tregs, whereas cluster 1 showed enriched of antitumor immune cells, such as B cell naïve, mast cell activated, or monocyte. This observation, to some extent, elucidates why patients in the C2 group experienced greater benefits from immunotherapy but had a lower survival rate. The implications are twofold: first, the correlation of PCD genes with the TME deepens our understanding of tumor immunology in LIHC. Second, it advocates for the potential benefit of immunotherapy, especially in patients categorized under the C2 group.

The culmination of our analysis was the establishment of a PCD index (PCDI) for predicting patient prognosis. A robust predictive model was constructed using 17 PCD genes, showcasing a potential prognostic tool for LIHC. Notably, high PCDI was associated with pathways concerning treatment resistance and tumor invasion. This provides potential explanations for the worsened prognosis seen in patients with a high PCDI. Furthermore, the association between PCDI and immune landscapes, particularly the diminished immune responses in patients with high PCDI and the negative correlation with activated mast cells, sheds light on the immune evasion mechanisms at play. Previous study has proved a connection between tumor-infiltrating mast cells and resistance to anti-PD-1 therapy [39]. This connection further underscores the intricate interplay between PCD and the immune microenvironment, suggesting the potential for combined therapeutic strategies targeting both these aspects in LIHC. Meanwhile, our exploration into pharmacotherapeutic approaches offers promise. By correlating PCD gene expression with drug sensitivities, we’ve identified drugs that might be more efficacious in specific patient subgroups, potentially guiding personalized therapy. In comparison to previous studies, our research has provided a more in-depth understanding of the association between PCD and the immune microenvironment. In comparison with previously published prognosis models [40], we conducted a comprehensive assessment of the intrinsic heterogeneity among patients with high and low PCD levels, highlighting their varying sensitivity to immunotherapy. Furthermore, we have identified personalized therapeutic strategies for drug treatment based on these insights. Lastly, we verified that PRKDC, seldom mentioned in prior cancer studies, correlated with poorer survival outcomes in LIHC patients [24]. Furthermore, elevated PRKDC levels notably enhanced the proliferation and invasive capabilities of LIHC, suggesting it as a potential therapeutic target for this condition.

While the PCD model demonstrates strong predictive capabilities and PRKDC plays a key role in the resistance of LIHC cells to various treatments, including chemotherapy and targeted therapies, as well as in creating an immunosuppressive environment that hinders anti-tumor immune response, it is important to acknowledge certain limitations in this study. The ability of the PCDI to forecast outcomes of immunotherapy is initially based on estimates generated by the submap algorithm, and the precision of the PCDI requires validation using real-world LIHC immunotherapy datasets. Additionally, it is essential to conduct further *in vivo* experiments to elucidate the molecular mechanisms that connect PRKDC with tumor progression.

## CONCLUSIONS

Collectively, this comprehensive study delineates the profound interplay between programmed cell death genes, the immune landscape, and liver cancer prognosis. By identifying crucial genes and pathways, categorizing patient subgroups, and suggesting promising therapeutic approaches, our results inform a roadmap for future research and therapeutic interventions in LIHC.

## Supplementary Material

Supplementary Figures
