# Supplementary material for: Targeting PRKDC activates the efficacy of antitumor immunity while sensitizing to chemotherapy and targeted therapy in liver hepatocellular carcinoma
Source: Aging (Albany NY). 2024 May 22;16(10):9047–71. doi: 10.18632/aging.205855 (PMC11164487; doi:10.18632/aging.205855)
Supplement: Supplementary Figures [file aging-16-205855-s001.pdf]

SUPPLEMENTARY FIGURES

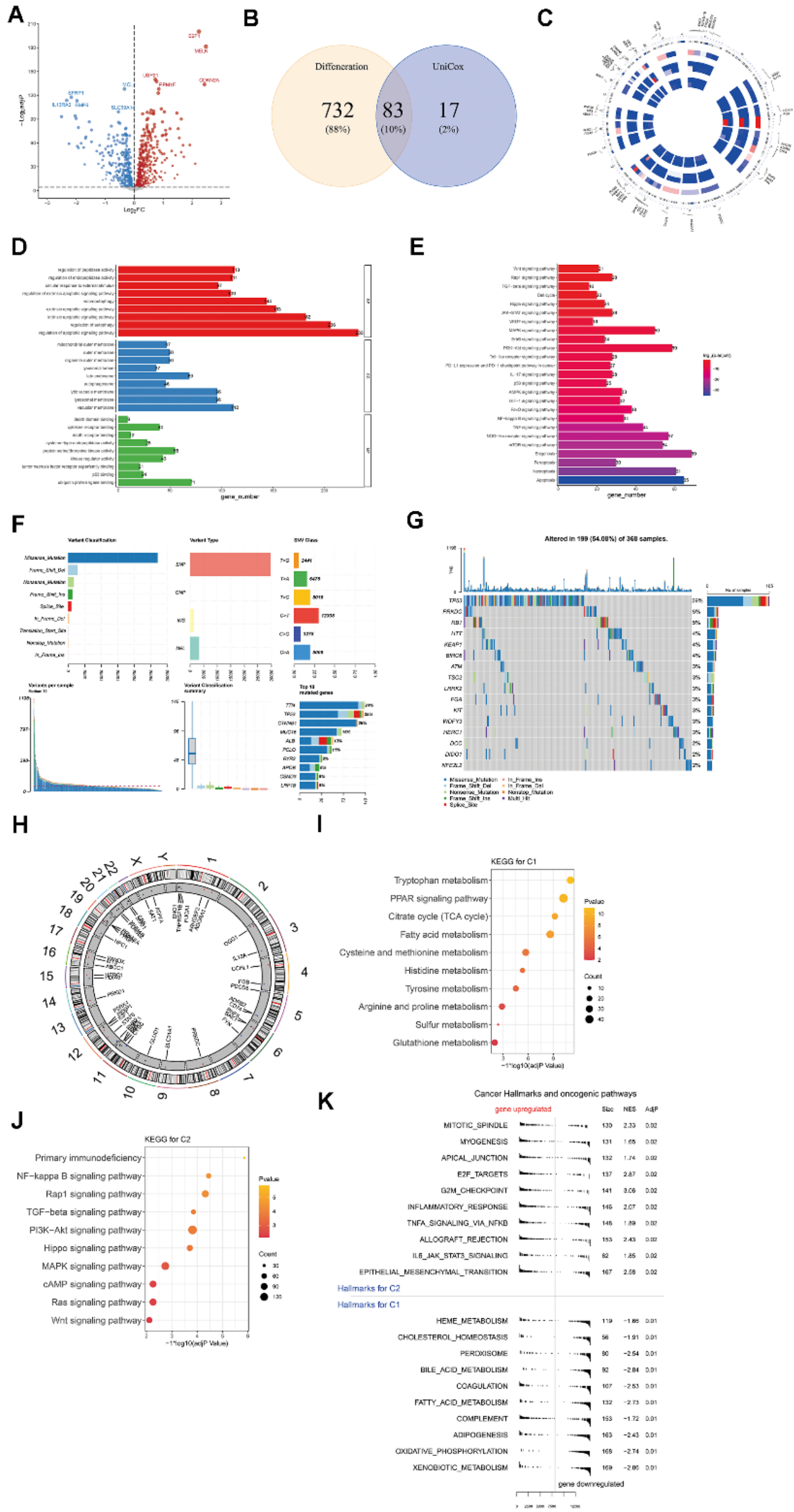

Supplementary Figure 1. Profiles of programmed cell death (PCD) genes in LIHC patients. (**A**) A volcano plot illustrates the expression differences of PCD-related genes in LIHC, with blue representing down-regulated genes, red for up-regulated genes, and grey for

those without significant change. (**B**) A Venn diagram depicts the intersection of PCD-related DEGs and those linked to patient outcomes. (**C**) A Circos plot presents the genomic location and expression of 45 PCD genes across five datasets. (**D**) GO analysis of PCD-associated DEGs. (**E**) KEGG analysis centered on PCD-related DEGs. (**F**) Summary of mutation landscape in the TCGA-LIHC dataset. (**G**) Oncoplot illustrating genes associated with PCD in the TCGA-LIHC dataset. (**H**) Circos plot demonstrate the CNV of selected 45 PCD genes on the genome in TCGA-LIHC. (**I**–**J**) Dot plot presents the results of KEGG enrichment analysis for both clusters. (**K**) Gene set enrichment analysis (GSEA) highlights hallmark pathways within each cluster.

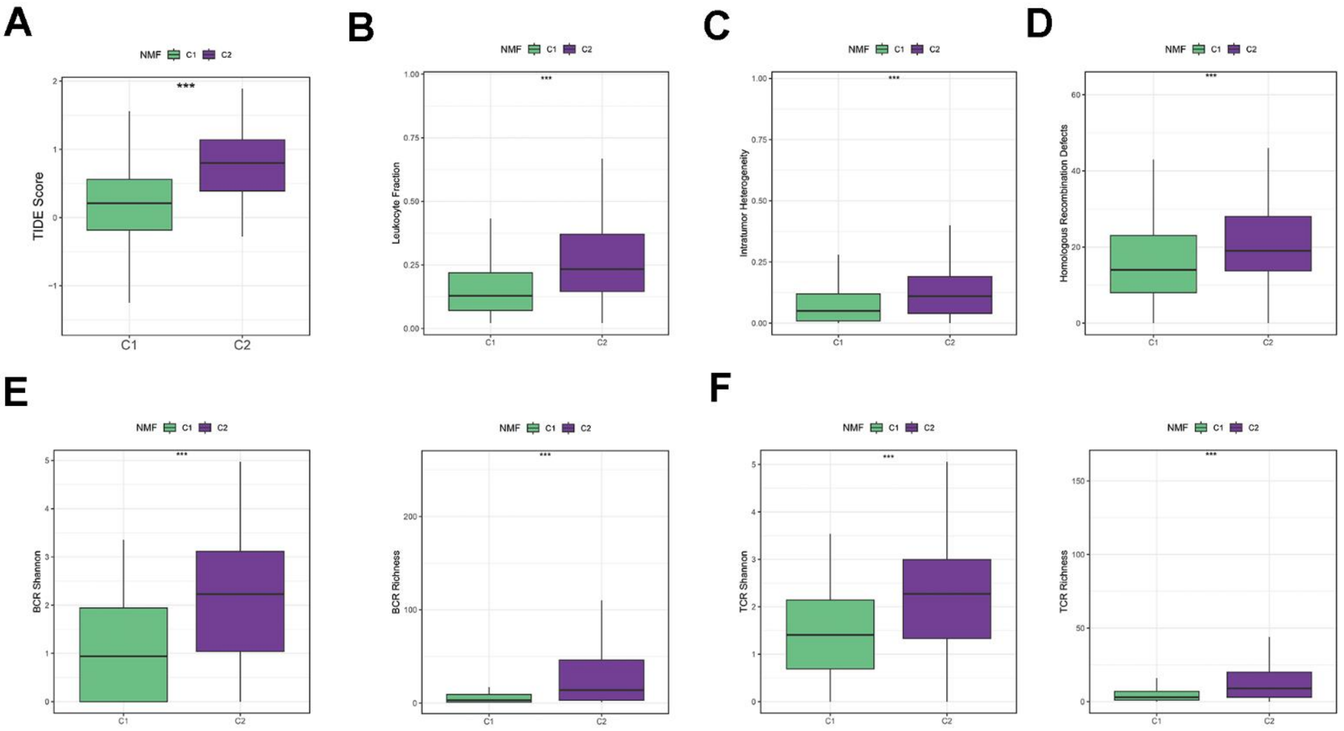

Supplementary Figure 2. Somatic diversification of two groups. (**A**) Comparative box plots contrasting Tumor Immune Dysfunction and Exclusion (TIDE) scores between C1 and C2. (**B**) Box plot illustrating normalized fraction level of leukocyte in C1 and C2. (**C**) Box plot showing the normalized score of intratumor heterogeneity between C1 and C2. (**D**) Box plot depicting the normalized score of homologous recombination defects in C1 versus C2. (**E**) Box plot presenting normalized score of BCR Shannon and Richness between C1 and C2. (**F**) Box plot contrasting normalized score of TCR Shannon and richness in C1 versus C2. (*$P < 0.05$, **$P < 0.01$, and ***$P < 0.001$ determined by two-way ANOVA test).

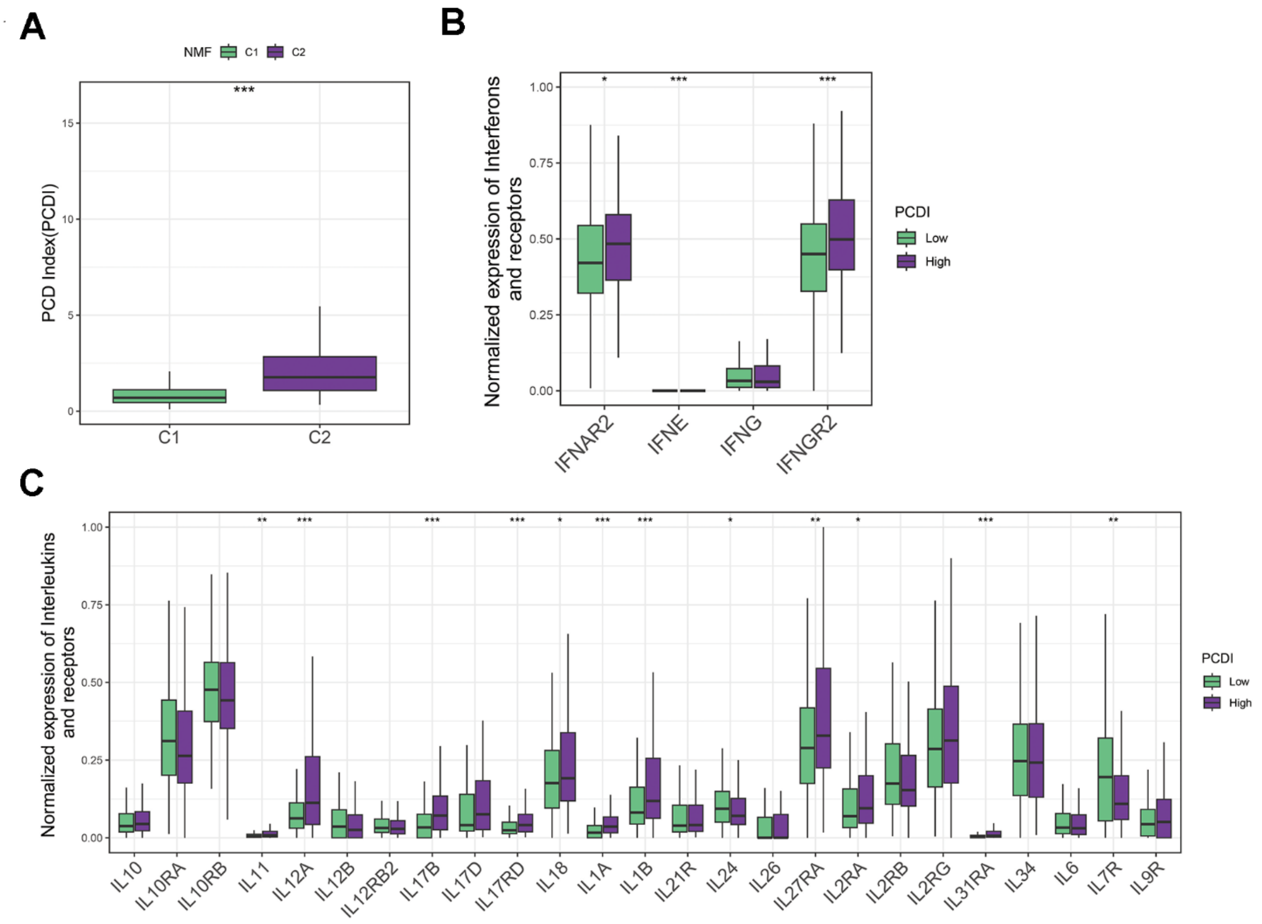

Supplementary Figure 3. Immune landscape of high and low PCDI groups. (**A**) Comparative box plots demonstrating PCDI in C1 and C2 groups. (**B**) Comparative box plots showcasing normalized expression levels of interferons and their receptors in two groups. (**C**) Comparative box plots demonstrating normalized expression levels of interleukins and receptors in two groups. (*$P < 0.05$, **$P < 0.01$, and ***$P < 0.001$ determined by Wilcoxon test).

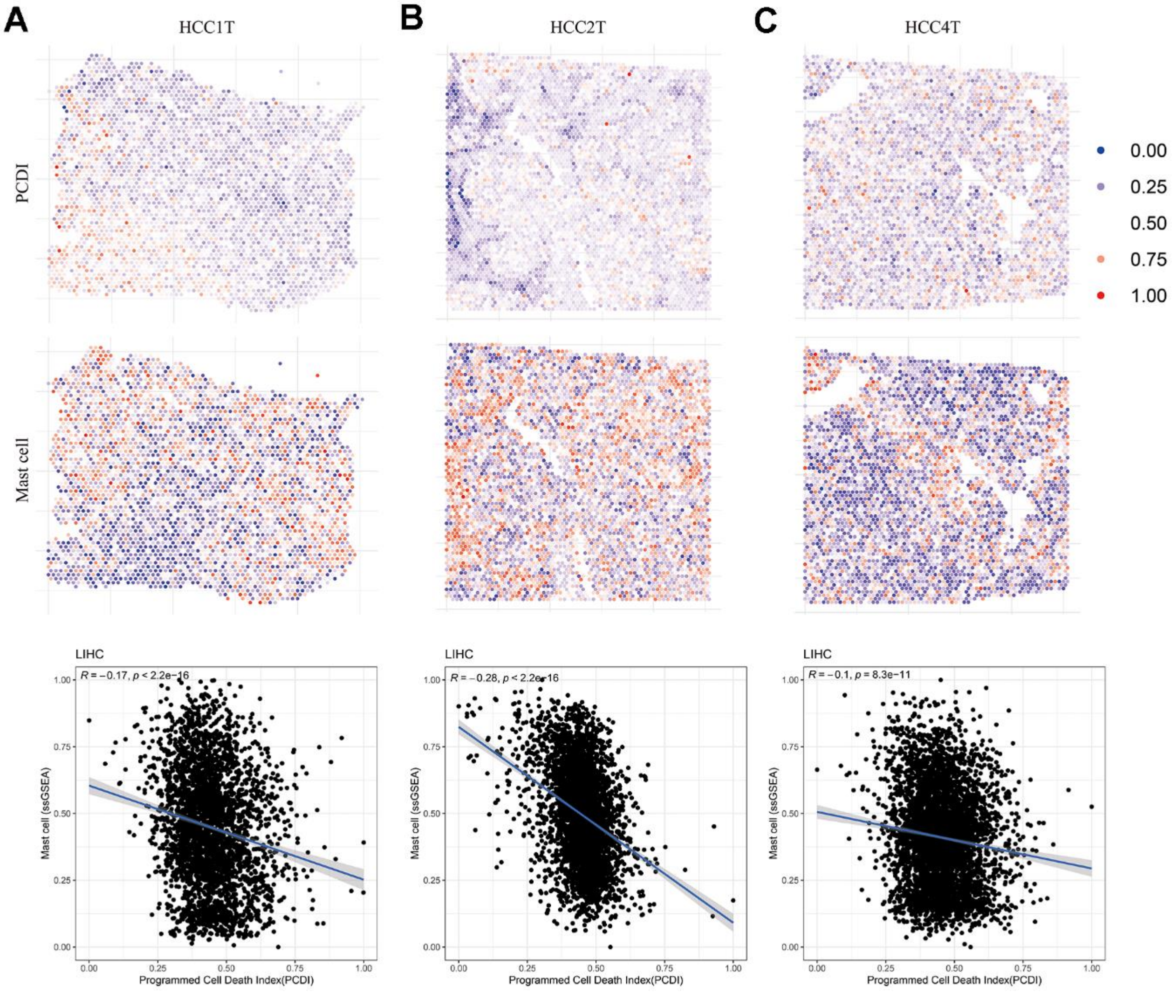

Supplementary Figure 4. Immune landscape of PCDI in two-dimensional spatial level. (**A**) PCDI score of each spatial plot in three LIHC spatial transcriptome samples. (**B**) GSVA score of mast cell of each spatial plots in three LIHC spatial transcriptome samples. (**C**) Correlation between mast cell score and PCDI in LIHC spatial transcriptome samples.

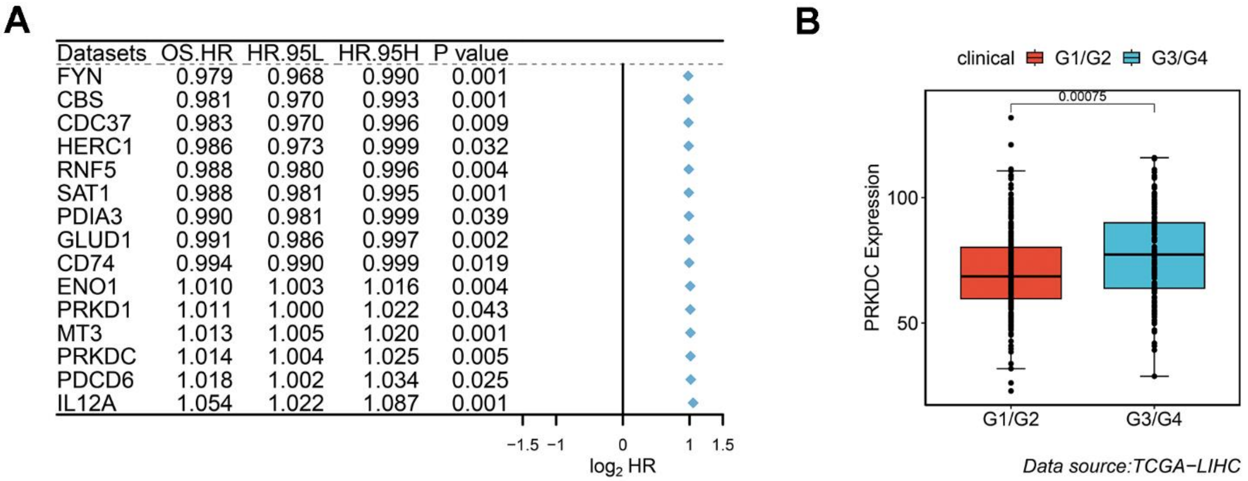

Supplementary Figure 5. Clinical correlation of PCD genes. (**A**) Univariate Cox regression analysis of PCD associated genes in PCDI. (**B**) The expression of PRKDC in G1/G2 and G3/G4 stage.
